# Immobilization of Thiol-Modified Horseradish Peroxidase on Gold Nanoparticles Enhances Enzyme Stability and Prevents Proteolytic Digestion

**DOI:** 10.1021/acs.langmuir.4c01180

**Published:** 2024-06-26

**Authors:** Faith E. Breausche, Annelise Somerlot, Jason Walder, Kwame Osei, Samuel Okyem, Jeremy D. Driskell

**Affiliations:** † Department of Chemistry, Illinois State University, Normal, Illinois 61790, United States; ‡ Department of Chemistry, University of Illinois at Urbana−Champaign, Urbana, Illinois 61801, United States

## Abstract

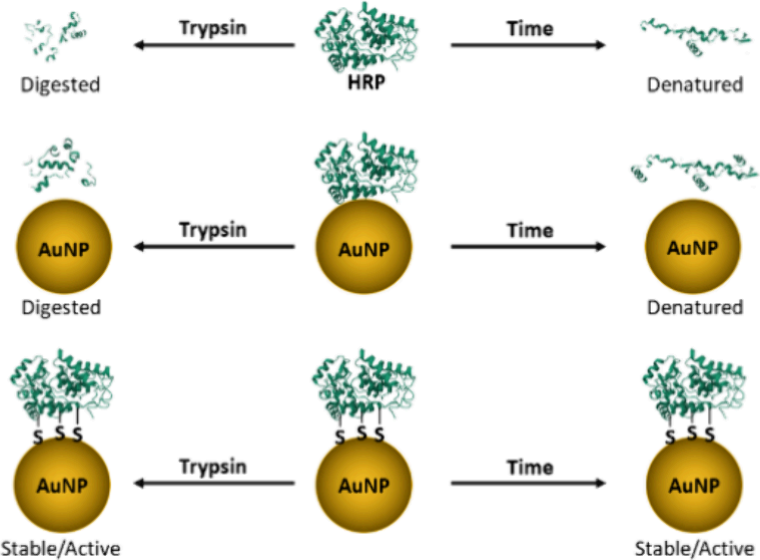

The specificity and efficiency of enzyme-mediated reactions have the potential to positively impact many biotechnologies; however, many enzymes are easily degraded. Immobilization on a solid support has recently been explored to improve enzyme stability. This study aims to gain insights and facilitate enzyme adsorption onto gold nanoparticles (AuNPs) to form a stable bioconjugate through the installation of thiol functional groups that alter the protein chemistry. In specific, the model enzyme, horseradish peroxidase (HRP), is thiolated via Traut’s reagent to increase the robustness and enzymatic activity of the bioconjugate. This study compares HRP and its thiolated analog (THRP) to deduce the impact of thiolation and AuNP-immobilization on the enzyme activity and stability. HRP, THRP, and their corresponding bioconjugates, HRP-AuNP and THRP-AuNP, were analyzed via UV–vis spectrophotometry, circular dichroism, zeta potential, and enzyme–substrate kinetics assays. Our data show a 5-fold greater adsorption for THRP on the AuNP, in comparison to HRP, that translated to a 5-fold increase in the THRP-AuNP bioconjugate activity. The thiolated and immobilized HRP exhibited a substantial improvement in stability at elevated temperatures (50 °C) and storage times (1 month) relative to the native enzyme in solution. Moreover, HRP, THRP, and their bioconjugates were incubated with trypsin to assess the susceptibility to proteolytic digestion. Our results demonstrate that THRP-AuNP bioconjugates maintain full enzymatic activity after 18 h of incubation with trypsin, whereas free HRP, free THRP, and HRP-AuNP conjugates are rendered inactive by trypsin treatment. These results highlight the potential for protein modification and immobilization to substantially extend enzyme shelf life, resist protease digestion, and enhance biological function to realize enzyme-enabled biotechnologies.

## Introduction

Enzymes are proteins that catalyze biochemical reactions critical to many biological processes. Consequently, the high specificity and catalytic efficiency afforded by enzymes have been exploited for many non-native applications, such as diagnostics, chemical sensing, biosynthesis, and environmental monitoring/remediation.^1−3^ The susceptibility of enzymes to degradation in the harsh environment of these bionanotechnology applications, and the desire to recover enzymes in these systems, often requires immobilization of the enzyme on a solid support.^4^ Efforts to immobilize enzymes on flat supports have resulted in degraded performance due to slower diffusion rates, blocked active sites, and conformational changes that negatively impact function.^5^ Enzyme immobilization on nanoparticles, however, has resulted in examples of enhanced enzyme function, attributable to electrostatic forces between substrates and nanoparticles driving greater mass transport rates and increased surface area for greater enzyme loading.^6−10^

Gold nanoparticles (AuNPs) are promising carriers for enzymes. The size and shape of AuNPs are easily tuned, and the surface is readily modified to control charge, hydrophobicity, and chemistry. Moreover, it is well-established that proteins spontaneously adsorb to AuNPs through several forces, including electrostatic, hydrophobic, and Au–S interactions. Upon adsorption of the enzyme to the AuNP, it is critical that conformational changes do not impede function, the active site is not sterically blocked, and the interaction is robust enough to withstand desorption. Several enzyme-AuNP bioconjugates have been prepared and studied, with some immobilized enzymes exhibiting enhanced biocatalytic activity relative to the native enzyme^8,11−17^ and other enzymes resulting in a significant loss of function resulting from immobilization.^15,18−20^ Given these system-dependent outcomes, there is a substantial effort within the scientific community to identify the AuNP property (e.g., morphology, size, and surface chemistry) that is responsible for achieving maximum enzymatic activity for the bioconjugate.^8,16,21−28^

An electrostatic attraction between a protein and AuNP is responsible for the initial interaction between a protein and AuNP.^29−33^ It is on this premise that Tadepalli et al. investigated the influence of AuNP surface charge on the adsorption and catalytic activity of horseradish peroxidase (HRP).^23^ As anticipated, the surface charge was found to be a controlling factor in the structure, orientation, and activity of immobilized HRP and could be exploited to optimize the bioconjugate function. However, nanoparticle surface charge effects are enzyme- and pH-dependent. Moreover, protein adsorption is dynamic; once in contact, the protein can undergo AuNP-induced conformational changes to achieve a thermodynamically preferred interaction, which profoundly affects the biological function of the enzyme.^33−38^ The time to realize the final equilibrium state can take days.^39^ Consequently, studies that optimize AuNP properties for enzyme-AuNP bioconjugate performance are likely reflective of the initial protein-AuNP interaction and may have a short shelf life as the protein structure “evolves” to achieve a thermodynamically stable interaction at the interface. Control of the enzyme-AuNP interface is critical to attaining proper orientation and establishing a thermodynamically favorable interaction, without denaturation, to expose high-affinity interactions.

Here, as an alternative to investigating and manipulating AuNP properties, the enzyme-AuNP interaction is viewed through the lens of enzyme properties, which can be altered. Specifically, for AuNPs, proteins have been reported to structurally deform/rearrange to form the energetically preferred Au–S bond.^31,39−45^ Thus, an enzyme that presents solvent-accessible thiol functional groups would benefit from a thermodynamically favored interaction while maintaining its native conformation. Moreover, we hypothesize that the presence of multiple surface-accessible thiols to facilitate multipoint immobilization would provide added rigidity to enhance enzyme stability relative to the free enzyme in solution.^38,46−49^ In this work, HRP was used as a model enzyme to investigate the effect of surface-accessible thiols on the adsorption to gold nanoparticles. HRP was selected as the model enzyme because it has been previously conjugated to AuNPs,^8,23^ its enzyme–substrate activity is readily quantified spectrophotometrically, and it has been employed in many applications such as biosensing, bioremediation, and chemical synthesis.^50,51^ Important for this study, native HRP contains eight cysteine residues, all involved in disulfide bonds, thus limiting the possibility of Au–S interactions through an accessible cysteine side chain. A thiolated analog of HRP (THRP) was synthesized via chemical modification to convert the amine of a lysine residue side chain to a thiol using Traut’s reagent and subsequently confirmed to have equivalent structure and biocatalytic activity as the native HRP. We then systematically tested the hypothesis that installation of additional thiol functional groups on the HRP molecule will facilitate multipoint attachment, resulting in increased protection from denaturation, greater structural integrity, and enhanced biological activity for the immobilized THRP. These results are immediately relevant to HRP-enabled biotechnologies ranging from biosensing to bioremediation,^50,51^ yet we anticipate that the insights related to thiolation and immobilization of HRP on stability and activity can be generalized to other enzymes.

## Experimental Section

### Materials and Reagents

Horseradish peroxidase (HRP), 2,2′-azino-bis(3-ethylbenzothiazoline-6-sulfonic acid)-diammonium salt (1-Step ABTS), potassium phosphate monobasic, sodium hydroxide, hydrochloric acid, tris base, glycine, bromophenol blue, 2-iminothiolane (Traut’s reagent), immobilized trypsin, and Zeba desalting columns (MWCO 7K Da) were purchased from ThermoFisher Scientific. Sodium dodecyl sulfate (SDS) was purchased from Sigma. Precision Plus Protein Dual Color Standard was purchased from Bio-Rad. Potassium phosphate dibasic was purchased from Mallinckrodt Chemical. Citrate-capped gold nanoparticles (AuNPs) with a nominal diameter of 60 nm and concentration of 0.05 mg/mL (∼2.6 × 10^10^ NP/mL) were obtained from Cytodiagnostics and NanoComposix. Samples were prepared and stored in 1.7 mL low-binding microcentrifuge tubes (Costar), and kinetic reactions were performed and monitored in low-binding 96-well plates (Greiner Bio-One). All buffers and aqueous solutions were prepared with deionized water (18 MΩ) from a Barnstead water purification system, and chemicals were used as received without further purification.

### Thiolation of HRP with Traut’s Reagent

A 113 μM stock solution of HRP was prepared by dissolving 5.0 mg of HRP into 1.0 mL of 0.1 M phosphate buffer (pH 6) containing 1.0 mM EDTA. The 1.0 mL stock was divided into two 500 μL aliquots. To one aliquot, 10 μL of buffer was added, and to the other HRP aliquot, 30-molar excess of Traut’s reagent (10 μL at 170 mM in 0.1 M phosphate buffer with 1 mM EDTA at pH 6) was added, and the mixture was allowed to react at room temperature for 1 h to form the thiolated HRP analog (THRP). After the incubation, the THRP sample was purified using a 2 mL Zeba desalting column, following the manufacturer’s protocol. It should be noted that native HRP samples also underwent the same purification process to confirm that neither the spin column nor the centrifugation conditions denatured or otherwise negatively impacted the enzyme function. The final concentration of the purified HRP and THRP stock solutions was determined through UV–visible spectrophotometry and calculated with an extinction coefficient of 2.89 × 10^4^ M^–1^cm^–1^ (*E*_1%_ = 6.04) at 280 nm. The purified enzyme stocks were aliquoted into 10 μL and stored in the freezer.

### Mass Spectrometry of HRP and THRP

MALDI-TOF mass spectrometry (MS) was conducted to measure the molecular weights of HRP and THRP. HRP and THRP samples were mixed with an equal volume of a saturated solution of sinapinic acid, prepared in a mixture of 30:70 v/v acetonitrile:0.1% TFA in water. One μL of each mixture was then deposited onto an MTP ground steel MALDI target for MS analysis. The smart beam laser was set to 4_large. MS data were acquired within an *m*/*z* range of 10 to 70 kDa, by accumulating 1000 laser shots. An optimized MS setting was employed using 20, 18.5, and 8.5 kV for ion sources 1, 2, and lens, respectively.

### Enzyme Activity Assay

The catalytic function of HRP and THRP was quantified by the enzyme-mediated oxidation of ABTS. A 10 μL aliquot of the sample (e.g., HRP, THRP, HRP-AuNP, or THRP-AuNP) was placed in a low-binding 96-well plate and gently mixed with 150 μL of a room-temperature one-step ABTS solution. The absorbance of the colored product was measured at 10 s intervals for a total of 10 min at 410 nm. The enzyme activity was defined as the average reaction rate calculated from the first 10 min of the reaction in units of OD/min. Each sample was plated in duplicate, and the enzyme activities are reported as average reaction rates.

### Preparation of Enzyme-AuNP Bioconjugates

HRP or THRP was passively adsorbed onto gold nanoparticles to form protein-AuNP bioconjugates, as previously described.^8,23,45,52^ Gold nanoparticles (100 μL) were pipetted into a low-binding microcentrifuge tube and centrifuged at 5000*g* for 5 min. The supernatant (90 μL) was removed from the pelleted AuNPs and replaced with 90 μL of 2 mM phosphate buffer (pH 6.0). After this buffer exchange, 1–10 μL of HRP or THRP solution (10 or 100 μg/mL) was added to introduce the desired amount of enzyme with final concentrations of the enzyme ranging from 0.1 to 10 μg/mL. The contents were gently mixed via a micropipette and left to rest at room temperature for 1 h to allow for the enzyme to adsorb. The conjugates were centrifuged three times for 5 min at 5000*g*. In between each centrifugation cycle, the colorless supernatant was removed and replaced with an equivalent volume of 2 mM phosphate buffer (pH 6.0). A purified enzyme-AuNP conjugate was produced after the third centrifugation/resuspension step.

### Stability of Enzyme-AuNP Bioconjugates

Enzyme-AuNP conjugates were prepared by the incubation of AuNP with 10 μg/mL HRP or THRP to achieve maximum enzyme loading on the AuNP surface. The conjugates were then purified, as described above. Solutions of HRP and THRP were prepared at 0.5 μg/mL in a 2 mM phosphate buffer (pH 6.0). The enzyme activities of the four samples (e.g., HRP, THRP, HRP-AuNP, and THRP-AuNP) were initially measured to establish the baseline activity for the freshly prepared samples. Each of the four samples was divided into three aliquots. One aliquot of each sample was stored at a different temperature, 4 °C, room temperature (e.g., 22 °C), or 50 °C. The activity of each sample stored at each temperature was determined after 6 h, 24 h, 3 days, 1 week, and 1 month to evaluate the stability of the enzymes with respect to storage time. The activity of each sample was compared to its initial activity to calculate the fraction of the remaining activity at each time point. At each time point, the samples were plated in triplicate to calculate the average enzyme activity. The entire stability study was performed on three separate occasions, and the results of the independent experiments were averaged.

### Instrumentation

Mass spectrometry (MS) analysis was conducted using a Bruker UltrafleXtreme MALDI-TOF/TOF instrument (Bruker Daltonics, Billerica, MA) operating in positive linear mode and equipped with a proprietary smart beam laser. The MS was calibrated using Protein Standard II (Bruker Daltonics). The absorbance spectra of HRP and THRP were acquired using a Cary 3500 UV–visible spectrophotometer (Agilent) to evaluate the impact of chemical modification manifested as spectral shifts and calculate enzyme concentrations. The Cary 3500 was also used to collect the extinction spectra of unconjugated AuNP, HRP-AuNP, and THRP-AuNP to confirm the protein adsorption onto AuNPs. A Jasco J-1100 circular dichroism spectrophotometer was used to analyze HRP and THRP for differences in the secondary structure. Hydrodynamic diameter and zeta potential of the unconjugated AuNP, HRP-AuNP conjugates, and THRP-AuNP conjugates were measured by using a Zetasizer Nano ZSP (Malvern Panalytical). All enzyme activity assays performed in 96-well plates were analyzed with a Varioskan LUX multimode microplate reader operating in absorbance mode. A GelDoc-It^2^ (Analytik Jena) was used to acquire images and visualize protein bands in SDS-PAGE gels.

## Results and Discussion

### Synthesis and Characterization of Thiolated HRP Analog (THRP)

Horseradish peroxidase (HRP) is an excellent enzyme model to probe the influence of thiols on the structure and function of enzymes upon adsorption onto gold nanoparticles. HRP is a single-chain polypeptide with 308 amino acid residues, including 8 cysteine residues that are all involved in disulfide bridging. These cysteines are relatively solvent inaccessible; thus, it is unlikely that they interact with the AuNP unless the protein denatures. The solvent-accessible lysine residues on HRP can be exploited to install free thiols on HRP using amine-reactive chemistry to synthesize a thiolated HRP analog (THRP).^45,48,52,53^ Of the six lysine residues in HRP, it was previously established that three have side chains that are readily reactive via the primary amine (Lys174, Lys232, and Lys241), while the other three lysine residues are unavailable for modification because they are not exposed (Lys84), or sterically hindered by ion pairing and glycans (Lys65 and Lys149).^54^

**Figure 1:**
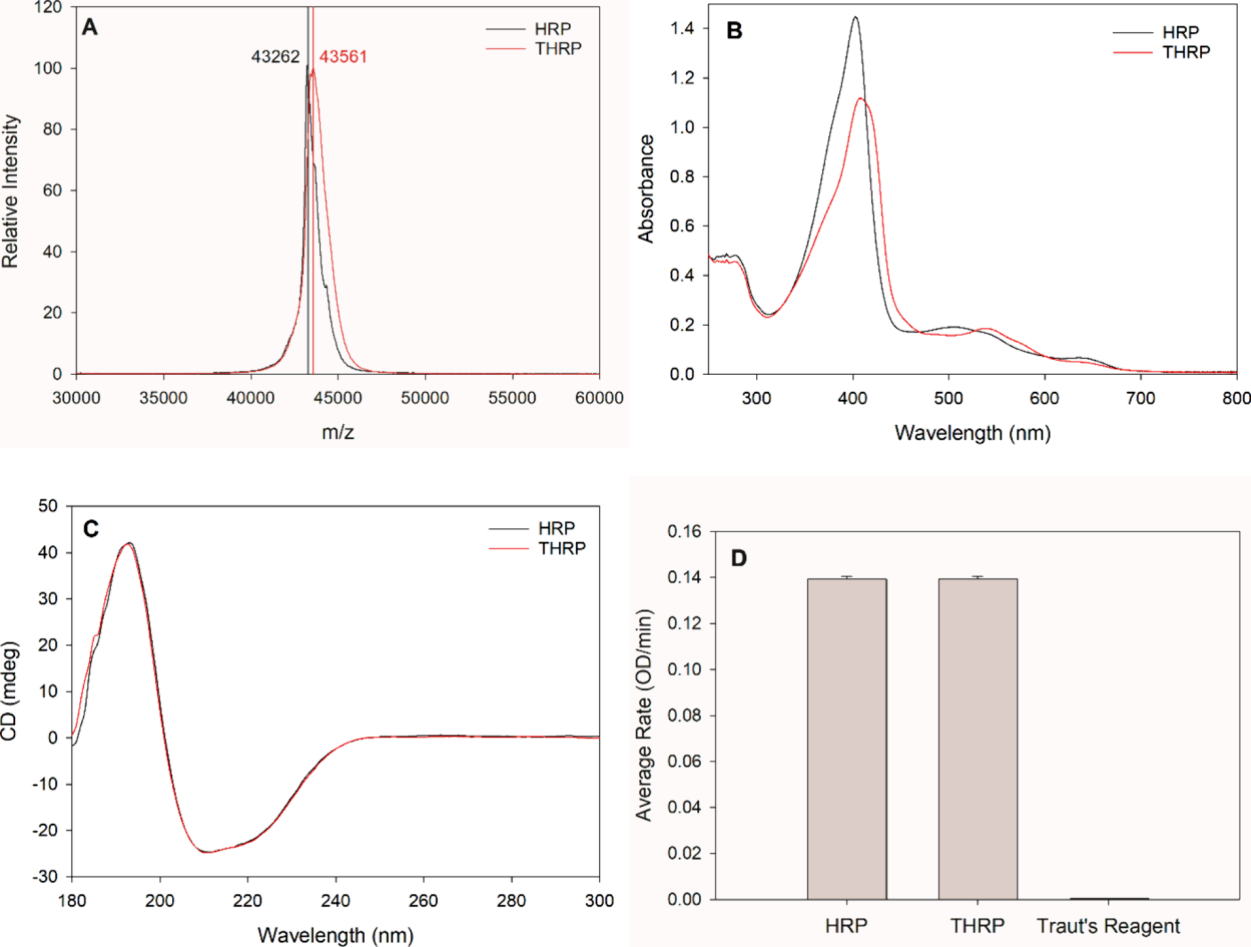
Characterization of HRP and THRP by MALDI-TOF mass spectrometry (A), UV–vis absorbance (B), circular dichroism (C), and enzyme activity (D). The enzyme activity is measured as the reaction rate for the oxidation of 1-step ABTS, and the error bars represent the standard deviation (*N* = 3).

HRP was mixed with Traut’s reagent to chemically install free thiol functional groups onto accessible lysine residues of HRP, generating the THRP species. HRP and THRP were analyzed by MALDI-TOF mass spectrometry to confirm Traut’s reagent effectively modified HRP (Figure 1A) and SDS-PAGE under nonreducing conditions to confirm that installation of the thiol did not induce unwanted protein aggregation via disulfide bridging (Supporting Information; Figure S1). Peaks for the singly charged HRP and THRP samples are observed at 43,262 and 43,561 Da, respectively, as expected for the glycosylated protein (Figure 1A).^55^ A 100.2 Da increase in molecular weight is expected for each lysine residue modified by Traut’s reagent. Thus, based on the experimentally measured difference in HRP and THRP masses (e.g., 299 Da), the data confirm that three lysine residues were modified and are consistent with previous reports detailing the accessibility of three lysine residues in HRP for chemical modification.^54^

HRP and THRP were spectrophotometrically characterized with UV–vis absorbance and circular dichroism (CD). HRP exhibits peak absorbances characteristic of the Soret band at 403 nm and Q-band at 505 mm (Figure 1B)**.** These peak absorbances have been previously established for the enzyme in its resting state in which the iron in the heme center exists as Fe^III^.^55,56^ A slight red shift in the Soret band to 408 nm and the Q-band to 537 nm is measured for THRP (Figure 1B). These spectral shifts are attributed to minor changes in the local environment around the iron at the heme center of HRP and are evidence of successful modification of HRP via Traut’s reagent. The CD spectrum reveals that HRP is predominantly α-helical in secondary structure, consistent with its well-established structure (Figure 1C).^55^ Furthermore, the CD spectrum of THRP is nearly indistinguishable from that of HRP, confirming that Traut’s modification did not alter the secondary structure of the enzyme.

An enzymatic assay was performed to assess the impact of chemical modification on the HRP function. The enzyme-catalyzed oxidation rate of ABTS is highly sensitive to enzyme concentration, and quantitation of the enzyme stocks is difficult to accurately determine spectrophotometrically because of slight shifts in the absorbance spectra of THRP, as discussed above. To address this challenge, one HRP stock solution was prepared at 0.5 μg/mL and split into two aliquots. To one 500 μL aliquot was added 10 μL of Traut’s reagent, and to the second 500 μL aliquot, 10 μL of buffer was added. After 1 h of incubation to allow for chemical modification, an enzyme activity assay was performed for both samples to evaluate the impact of thiolation on enzyme activity. HRP and THRP kinetic analyses were performed without purification to avoid challenges related to different concentrations based on the recovery variance through a desalting column. Kinetic analysis of a control sample, 10 μL of Traut’s reagent added to 500 μL of buffer, was also performed to ensure that excess Traut’s reagent in the THRP sample did not alter the enzymatic reaction rate. Figure 1D clearly shows equivalent enzymatic activity for HRP and THRP and no detectable activity for the Traut’s reagent negative control sample. Collectively, these data establish that modification of HRP by Traut’s reagent does not alter the enzyme structure or catalytic function.

### Enzyme Adsorption onto Gold Nanoparticles

**Figure 2:**
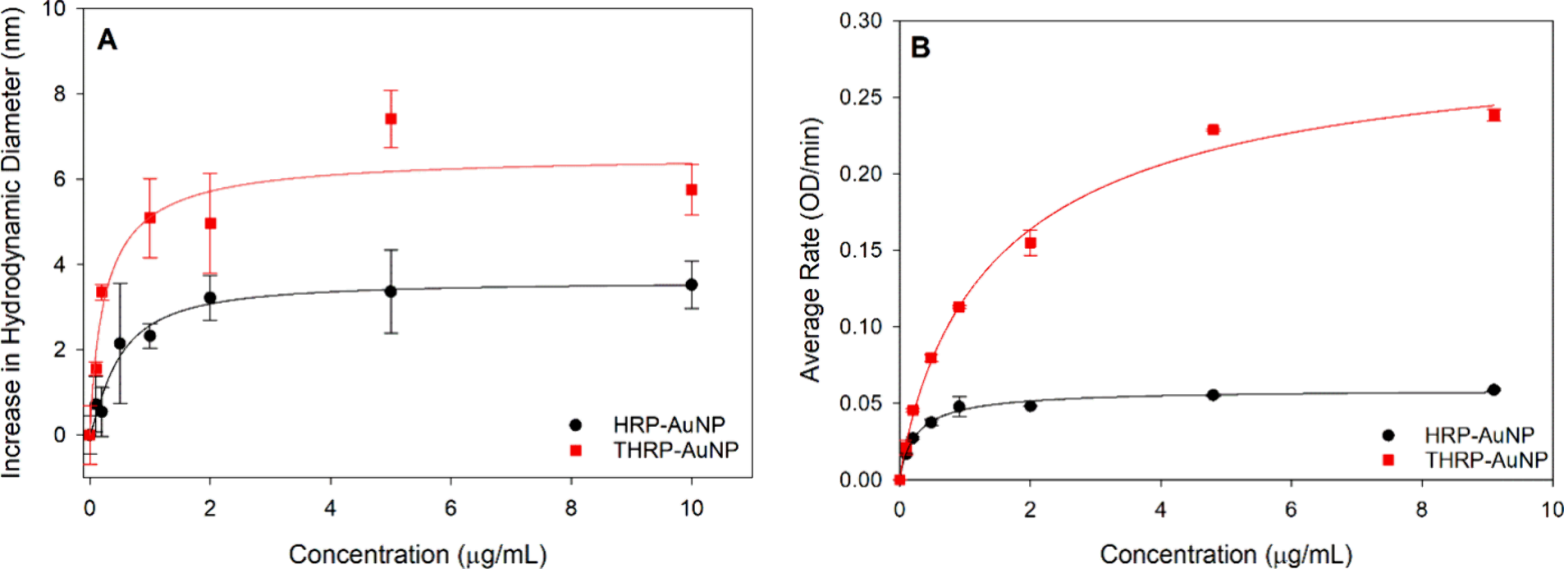
Adsorption isotherms for the spontaneous interaction of HRP and THRP with AuNP. Enzyme adsorption was monitored as the growth in the hydrodynamic diameter for the AuNP as a function of enzyme concentration using dynamic light scattering (A). Bioconjugate enzymatic activity was measured for conjugates prepared with increasing concentrations of enzyme (B). Analysis includes two independent preparations of each sample with three DLS size measurements for each sample and duplicate enzyme–substrate reactions for each sample. All measurements were averaged, and standard deviations are represented by the error bars.

Gold nanoparticles were incubated with HRP or THRP to investigate the effect of thiolation on the adsorption behavior. Increasing concentrations of each enzyme were mixed with a constant concentration of AuNPs for 1 h at pH 6.0 to allow for spontaneous adsorption of the enzyme onto the AuNP. Enzyme adsorption was monitored as an increase in the hydrodynamic diameter of the AuNP using dynamic light scattering (DLS). The adsorption isotherm in Figure 2A establishes that both HRP and THRP spontaneously adsorb to reach saturated coverage at saturation. The saturated HRP-AuNP and THRP-AuNP bioconjugates increase in diameter by 3.6 and 6.60 nm, respectively, relative to the unconjugated AuNP. These measured increases in diameter at maximum coverage are consistent with a previously measured value for an adsorbed layer of HRP on the AuNP and the dimensions of HRP (3.0 × 6.5 × 7.5 nm^3^).^57^ The difference in the HRP and THRP layer thicknesses suggests differences in protein orientation, loading, or structure (denatured/deformed) when adsorbed onto the AuNP.

Purified bioconjugates prepared with increasing amounts of HRP or THRP were then evaluated for enzymatic activity (Figure 2B). After incubating the AuNP with increasing amounts of enzyme for 1 h, the enzyme-AuNP mixtures were washed via centrifugation cycles to remove excess, unbound enzyme and to isolate the bioconjugates. The enzyme-catalyzed reaction rate of the HRP-AuNP conjugates initially increases as the concentration of HRP used to form the conjugates increases; the reaction rate then plateaus, reaching a maximum rate of ∼0.05 OD/min. A similar correlation between the concentration of THRP used to form the conjugate and reaction rate is observed for the THRP-AuNP bioconjugate formation; however, the maximum achievable reaction rate for the THRP-AuNP conjugate is approximately 5× greater than that of the HRP-AuNP conjugate. The data in Figure 2B and additional analysis of the enzyme present in the supernatant from sequential wash cycles (Figure S2) confirm that both HRP and THRP spontaneously adsorb onto AuNPs to form a robust layer that resists desorption during the centrifugation process. Protein interaction with AuNPs has been extensively explored, and a combination of electrostatic, hydrophobic, and Au–S bonds via cysteine residues is responsible for the spontaneous adsorption of proteins onto AuNPs.^31,39−45^ HRP does not present any free thiol functional groups; thus, it is anticipated to adsorb through electrostatic or hydrophobic interactions, and these results are consistent with other published works.^8,23^ The installation of free thiols on THRP and its impact on AuNP adsorption have not been previously studied but is expected to yield strong adsorption of THRP onto the AuNPs. Similar to the DLS-generated adsorption isotherms, Figure 2B also confirms saturation of the enzyme layer on the AuNP, given the increase in the reaction rate that plateaus. Importantly, at high enzyme concentration, the reaction rate does not decrease, confirming that the immobilized enzyme is not overcrowded to inhibit enzyme–substrate binding.

We propose three hypotheses for the drastic difference in the activity of the enzyme-saturated HRP-AuNP and THRP-AuNP bioconjugates. First, greater activity of THRP-AuNP conjugates could result from greater THRP loading on the AuNP compared to HRP. At pH 6.0, the overall net charge on HRP and THRP is negative (pI ∼ 5.7), which would lead to protein–protein charge repulsion and low surface density on the AuNP surface. However, THRP binds to the AuNP through a stronger Au–S interaction than does HRP, allowing for tighter packing in the presence of lateral electrostatic repulsion between bound protein molecules. As noted, HRP and THRP likely interact with the AuNP through different amino acid residues to result in differences in the immobilized enzyme orientation. Thus, a second plausible explanation for the difference in activity of the bioconjugates is that the active site of HRP is sterically hindered to a greater extent than THRP. Protein adsorption onto nanoparticles can be dynamic in which, after an initial adsorption, the protein undergoes conformational changes to achieve the most thermodynamically stable interactions. It is likely that THRP initially adsorbs to the AuNP through surface-accessible thiol functional groups that are thermodynamically preferred, while the HRP molecules require slight deformation to achieve the most stable interaction. Thus, HRP structural changes (e.g., denaturation) induced by interaction with the AuNP are a third possible hypothesis for differences in bioconjugate activity.

**Figure 3:**
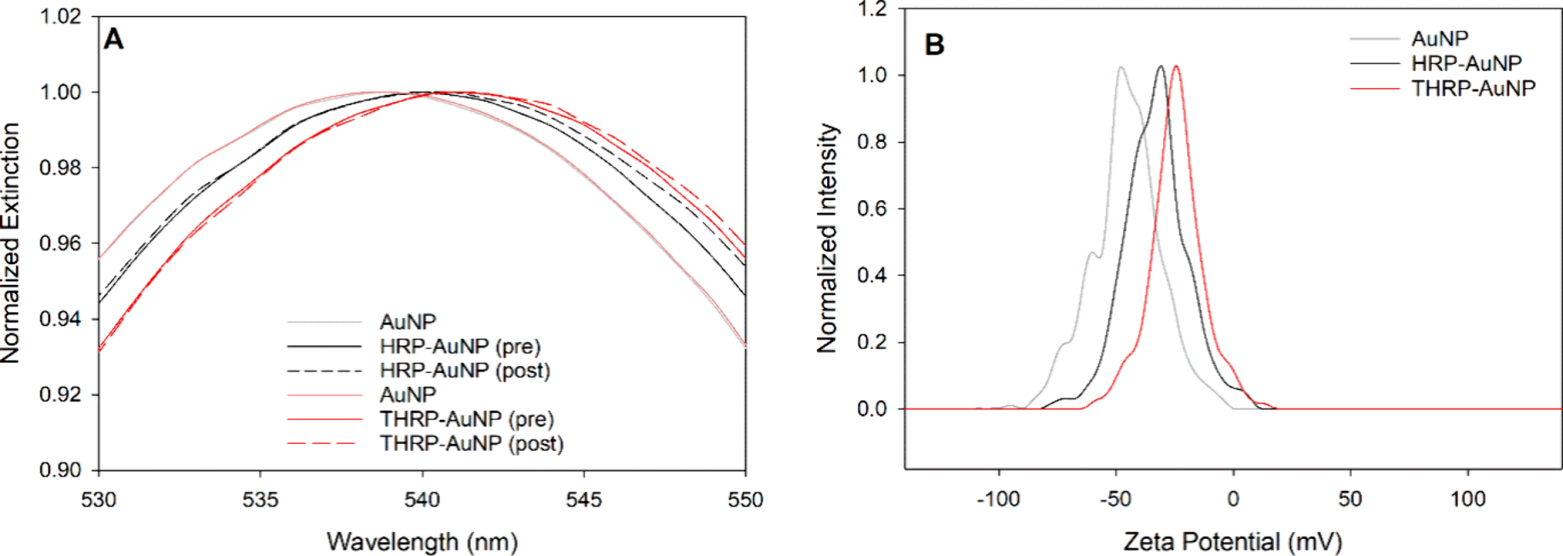
Characterization of fully saturated HRP-AuNP and THRP-AuNP bioconjugates with UV–visible extinction spectrophotometry (A). Enzyme was added to AuNPs at 10 μg/mL and incubated for 1 h. Bioconjugates were analyzed before purification, via centrifugation, and after purification to remove excess enzyme from the supernatant. Zeta potentials of the purified conjugates were then determined (B). An extinction spectrum and zeta potential measurement of the unconjugated AuNP were acquired as a comparative control sample.

Saturated bioconjugates were prepared and characterized to test these hypotheses and provide insights into different activities measured for HRP and THRP bioconjugates. AuNPs were incubated with HRP or THRP at sufficiently high concentrations (10 μg/mL) to ensure saturated coverage upon adsorption. Extinction spectra were collected for each conjugate prior to centrifugation to remove the unbound enzyme and compared to that of the unconjugated AuNP (Figure 3A). The localized surface plasmon resonance (LSPR) band red-shifted from 538 nm for the unconjugated AuNP to 540 nm for the HRP-AuNP conjugate and 541 nm for the THRP-AuNP conjugate. This 2–3 nm shift in LSPR resulted from a change in the local refractive index at the AuNP surface upon enzyme adsorption and is anticipated for enzyme adsorption onto AuNPs.^58,59^ The slightly larger shift observed for the THRP-AuNP conjugate indicates a thicker protein layer than the adsorbed HRP, and it corroborates the DLS data. No change in the LSPR was observed for the purified conjugates after centrifugation to remove the unbound enzyme from the suspension. These data confirm that both HRP and THRP are robustly immobilized onto the AuNP. A slight broadening of the extinction spectra at wavelengths greater than the LSPR after centrifugation may indicate the formation of a few aggregates that result from the harsh processing (e.g., centrifugation to pellet conjugates). However, any aggregate formation is quite minimal.

Zeta potentials of the unconjugated AuNP, purified HRP-AuNP bioconjugate, and purified THRP-AuNP bioconjugate measured −47.6, −31.1, and −24.5 mV, respectively (Figure 3B). The large negative charge of the unconjugated AuNP arises from the citrate capping agent, and it is well-established that the zeta potential increases (less negative value) as protein adsorbs onto citrate-capped AuNPs.^60^ Traut’s modification of lysine residues does not impact the overall protein charge, and the change in the AuNP zeta potential can be attributed to the quantity of the surface-adsorbed native or Traut’s-modified protein.^60^ Therefore, these zeta potentials qualitatively suggest that the increased enzyme activity of the THRP-AuNP bioconjugate compared to that of the HRP-AuNP bioconjugates is due to increased enzyme loading.

### Quantifying Enzyme Loading on Gold Nanoparticles

**Figure 4:**
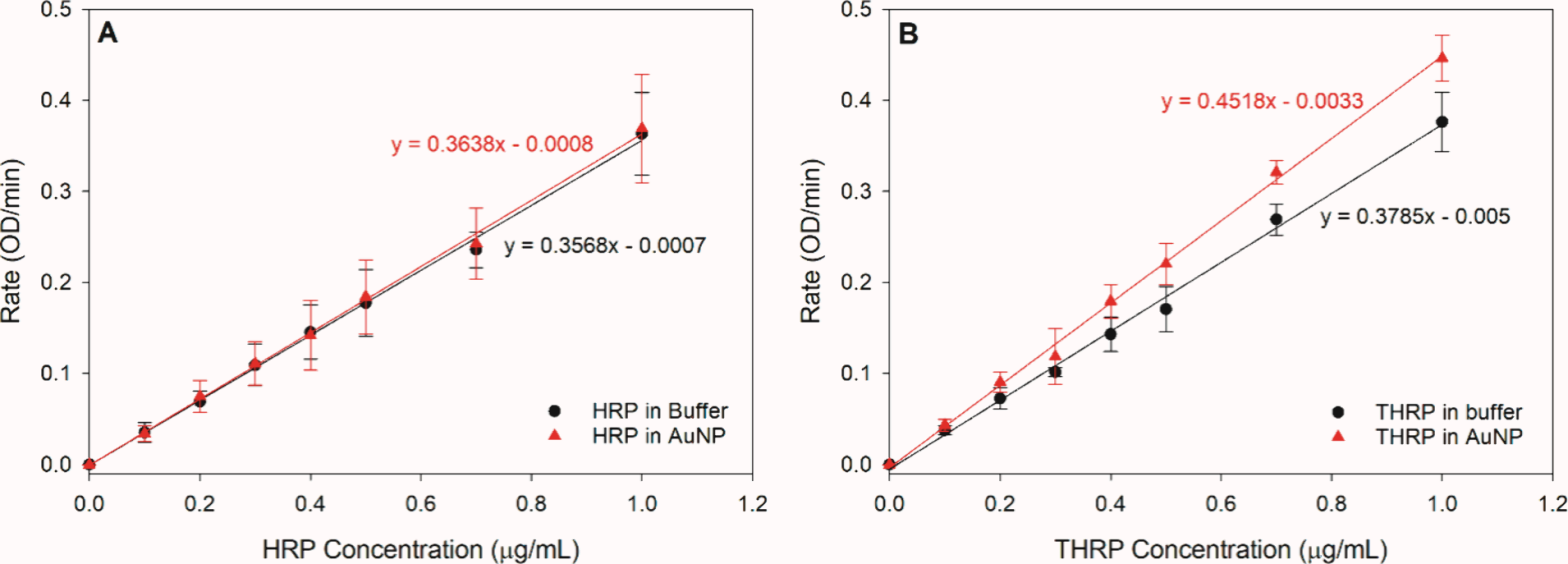
Calibration curves relating the enzymatic activity (e.g., reaction rate) of HRP (A) and THRP (B) to the enzyme concentration. Enzyme standard solutions were prepared in buffer or AuNP suspension as the solvent. Each standard solution was independently prepared five times by three unique individuals over a 6-month time period. The error bars represent the standard deviation.

Quantitation of HRP and THRP loaded onto a saturated conjugate was critical to compare the activity of the immobilized enzyme to that of the free enzyme, when normalized for concentration. Any reduced activity for the immobilized enzyme could be related to denaturation, unfavorable orientation, or diffusional effects. Moreover, immobilization consequences could be different for the native and thiolated analog of HRP. Protein loading is routinely quantified as the difference in the protein concentration added to form the conjugate and the concentration of protein remaining in the supernatant; however, this standard approach proved inaccurate because the enzyme adsorbed to the microcentrifuge tubes (Supporting Information; Figure S3). For this system, the enzymatic activity of HRP and THRP was exploited to quantify the adsorbed enzymes. We measured the activity of enzyme-AuNP bioconjugates and compared it to an external calibration curve relating the enzyme activity to varying concentrations of HRP and THRP in the solution. A major concern, however, was the potential inhibition of enzyme activity for the immobilized enzyme such that external calibration with standard solutions of free enzyme was not valid to quantify the immobilized enzyme on the bioconjugates. To determine if the enzyme–substrate reaction was affected by immobilization, calibration standards of HRP and THRP were prepared using buffer and the AuNP suspension as the diluent (Figure 4). The HRP activity (e.g., reaction rate) was equivalent for standard solutions prepared in a buffer and AuNPs (Figure 4A). As evidenced by Figure 2, a substantial amount of HRP adsorbed onto the AuNP in the concentration range of 0–1 μg/mL; therefore, the combined activity of the immobilized and unbound, free HRP in the AuNP diluent was equivalent to that of an equal concentration of free HRP in buffer. These data establish that free and AuNP-immobilized HRP activities are equivalent and that HRP loading on a saturated HRP-AuNP bioconjugate can be accurately quantified using the external calibration curve. Moreover, these results confirm that adsorption does not have detrimental effects on activity due to improper orientation or denaturation and identify differential enzyme loading as the cause for greater activity exhibited by the THRP-AuNP bioconjugates compared with the HRP-AuNP conjugates.

A linear correlation between enzyme activity and THRP concentration was also observed using both buffer and AuNPs as the diluents (Figure 4B). However, in contrast to the HRP calibration, a slight negative deviation was observed for the THRP standards prepared in buffer compared to THRP standards prepared in AuNPs. THRP had an affinity to adsorb to the microcentrifuge tube walls resulting in a loss of THRP molecules in the buffer; thus, the solution concentration was effectively lower than anticipated, causing a negative deviation in the reaction rate. When THRP standards were prepared in the AuNP matrix, the thiolated enzyme preferentially adsorbed onto the AuNP surface rather than the microcentrifuge tube walls, minimizing the loss of enzyme in suspension. This effect was not observed for the HRP calibration because HRP had a lower affinity for the microcentrifuge tube walls at these concentrations, limiting the loss of HRP through binding to the microcentrifuge tube walls (Figure S4). Collectively, these data suggest that THRP activity is not altered upon adsorption onto AuNPs, and THRP loading on bioconjugates can be accurately quantified via external calibration using AuNPs as the diluent.

To quantify enzyme loading on AuNPs, 10 μg/mL HRP or THRP was added to a suspension of AuNPs at pH 6.0. After allowing 1 h of adsorption to form a saturated layer on the AuNPs, the bioconjugates were purified via centrifugation and buffer replacement. The activity of the purified conjugates was recorded and compared to the calibration curves in Figure 4. Analysis of four independent preparations of bioconjugates resulted in the adsorption of 84 ± 42 HRP molecules per AuNP at saturation and 412 ± 38 THRP molecules per AuNP at saturation (Supporting Information). Assuming the smallest molecular footprint for HRP of ∼23 nm^2^ (3.0 × 6.5 nm^2^) and close molecular packing, a maximum of ∼500 HRP molecules per 60 nm AuNP could adsorb at monolayer coverage. Assuming a maximum molecular footprint of ∼49 nm^2^ (6.5 × 7.5 nm^2^), a close packed monolayer of HRP would result in ∼230 molecules adsorbed on each AuNP. The measured enzyme loading is reasonable using this geometric estimate and supports that THRP is most likely close packed, whereas the HRP molecules are more dispersedly packed on the AuNP surface.

### Effect of pH on HRP and THRP Adsorption onto AuNPs

The impact of solution pH during the spontaneous adsorption of the enzyme onto AuNPs was also investigated. AuNP suspensions were centrifuged, the supernatant was discarded, and the pelleted AuNPs were then dispersed in 2 mM buffers at pH 4.75, 6.0, 7.0, or 8.25. HRP or THRP was added to each pH-adjusted AuNP suspension at a concentration of 10 μg/mL and gently mixed. The resulting conjugates were incubated at room temperature for 1 h to ensure maximum adsorption. The bioconjugates were then purified via three centrifugation cycles with pellet resuspension in the respective pH buffers (e.g., pH 4.75, 6.0, 7.0, or 8.25). The HRP-AuNP and THRP-AuNP bioconjugates aggregated at pH 4.75. As noted previously, the pI of HRP is ∼5.7; thus, the enzymes are positively charged at pH 4.75 and induce AuNP aggregation via electrostatic bridging.^33,61,62^ Bioconjugates prepared at pH ≥ 6.0 are stable, and the protein has an overall net negative charge. It is worth noting that localized regions of positive charge are present on the HRP and THRP molecules even at pH > pI, which supports electrostatic attraction to the negatively charged AuNP. Enzymatic activity was then measured for each of the purified conjugates. The pH was held constant for the activity assays to ensure that any difference in activity was due to differences in the as-formed bioconjugates rather than differences related to pH-dependent enzyme–substrate reaction kinetics. The activity of the THRP-AuNP conjugates was ∼4× greater than the activity of the HRP-AuNP bioconjugates at each pH (Figure S5). The activity of both bioconjugates decreased as the pH increased. Using the calibration curves in Figure 4, the THRP loading decreased from a maximum of ∼411 THRP molecules per AuNP at pH 6.0 to ∼269 THRP molecules per AuNP at pH 8.25. The negative correlation between adsorbed molecules and pH is likely due to greater repulsive forces between the increasingly negative enzyme charge and the negatively charged AuNP. Additionally, as the net negative charge of each enzyme increases with pH, increased repulsion between neighboring immobilized enzymes results in a less densely packed protein layer. Similar pH-dependent behavior is observed for HRP where loading is maximized at pH 6.0 with ∼116 HRP molecules per AuNP and is reduced to ∼79 HRP molecules per AuNP at pH 8.25. Greater loading of THRP can be rationalized by the strong Au–S interaction that partially overcomes the electrostatic repulsive forces compared with the interactions responsible for HRP adsorption onto AuNPs.

### Stability of Free and Immobilized Enzymes

**Figure 5:**
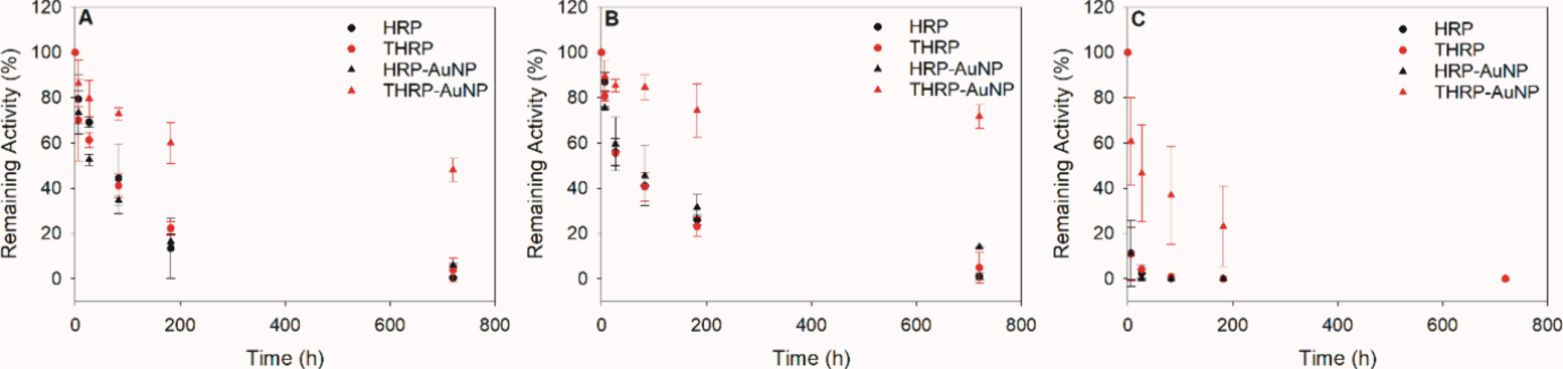
Stability of free and immobilized HRP and THRP with respect to time and temperature. Enzymatic reaction rates were determined for freshly prepared free enzymes, HRP and THRP, at 0.5 μg/mL and purified bioconjugates, HRP-AuNP and THRP-AuNP. Samples were stored at room temperature of 22 °C (A), 4 °C (B), or 50 °C (C). The activity of each sample was tested at various time points (1 h–1 month) and compared to its initial activity to assess stability. Three independent preparations of each sample were analyzed at each time point and temperature; the error bars represent the standard deviation.

The stability of the free and immobilized enzymes was systematically investigated with respect to time and temperature. Stock solutions of free HRP and free THRP were prepared at a concentration of 0.5 μg/mL in 2 mM buffer at pH 6.0 as the control samples. Purified HRP-AuNP and THRP-AuNP bioconjugates were also prepared with saturated levels of the enzyme at pH 6.0. An initial enzyme activity for each freshly prepared sample was recorded to define the maximum enzymatic activity. Each sample was then subdivided into three aliquots and stored at 4 °C, room temperature (∼22 °C), or 50 °C. Each sample was then tested for enzyme activity after different storage times (1 h–1 month) and compared to its initial activity to calculate the relative remaining activity as a function of storage time. At each temperature, the stability of the free enzymes and HRP-AuNP bioconjugates exhibited similar behaviors, while the activity of the THRP-AuNP bioconjugate degraded slower (Figure 5). For example, after 3 days at 22 °C, the free HRP, free THRP, and HRP-AuNP conjugates retained 44 ± 15%, 41 ± 5%, and 35 ± 2%, respectively, of their initial activities, whereas the THRP-AuNP conjugate retained 73 ± 3% of its initial activity (Figure 5A). After 3 weeks at 22 °C, only the THRP-AuNP bioconjugate exhibited enzymatic activity (48 ± 5%). No advantage was observed for storing free HRP, free THRP, or HRP-AuNP conjugates at 4 °C compared to room temperature within the first 3 days; however, a slight improvement in retained activity after 1 week of storage was observed at a lower temperature (Figure 5B). Notably, the THRP-AuNP bioconjugate maintained 72 ± 5% of its activity after 3 weeks at 4 °C. At an elevated temperature of 50 °C, no enzymatic activity was detected after 24 h for the free enzymes and the HRP-AuNP bioconjugate, yet the THRP-AuNP retained 23 ± 17% of its activity after 1 week (Figure 5C). This temperature-dependent trend in stability is expected; HRP should be stored at −20 °C to minimize denaturation and the loss of function. Interestingly, the stability of THRP was substantially enhanced upon immobilization onto AuNPs, while the immobilization of HRP onto AuNPs did not affect the stability relative to the free enzyme. There are several reports of enhanced enzyme stability upon immobilization on a solid support. The additional rigidity introduced by immobilization inhibits protein unfolding, which stabilizes its structure and function even at elevated temperatures. The free thiol functional groups on THRP act as high-affinity points of contact with the AuNP support to prevent unfolding and denaturation over time and at elevated temperatures. In contrast, HRP does not present free thiols, and its initial points of interaction with AuNPs are less robust, which, in concert with a less tightly packed HRP layer, the enzyme denatures and spreads on the AuNP surface over time to establish the most thermodynamically stable interaction, rendering it inactive. This unfolding/rearranging process can be relatively slow; the denaturation process for BSA on AuNPs was observed over a few days.^39^ Moreover, our group has previously reported that higher temperatures promote an increased denaturation rate of immobilized protein on AuNPs to establish a more robust interaction.^53^

### Proteolytic Degradation of Free and Immobilized Enzyme

**Figure 6:**
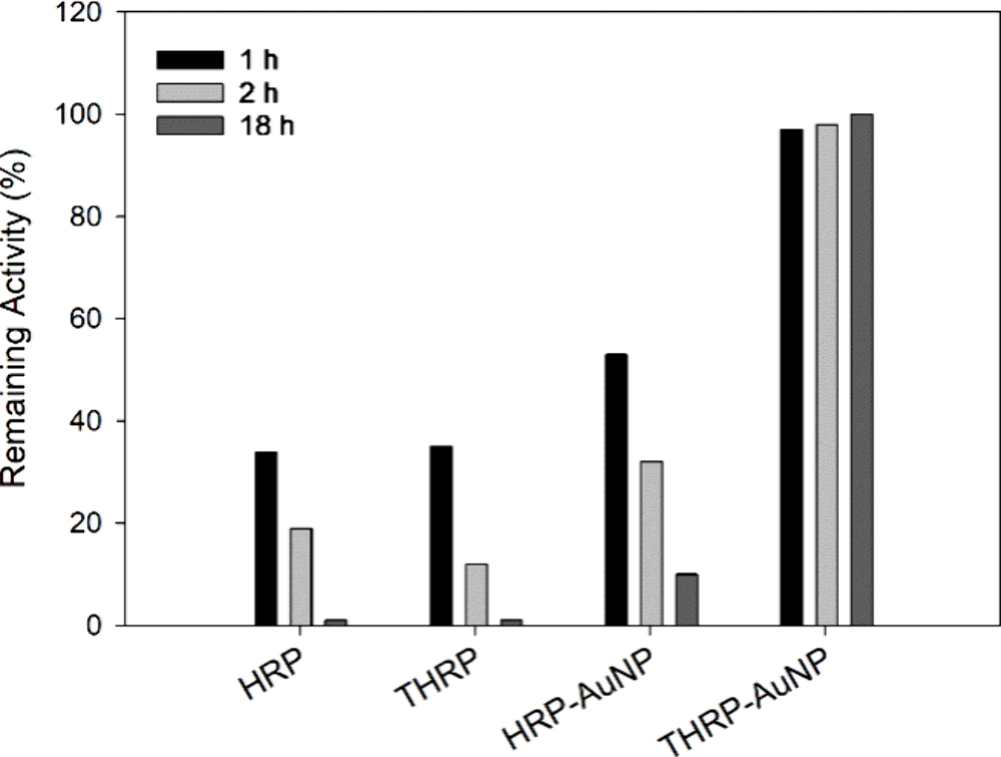
Susceptibility of free and immobilized HRP and THRP to trypsin digestion. Enzymatic reaction rates were determined for free enzymes, HRP and THRP, at 0.5 μg/mL and purified bioconjugates, HRP-AuNP and THRP-AuNP, after exposure to trypsin for 1, 2, and 18 h at 37 °C. Remaining activity is defined as the ratio of enzymatic activity for trypsin-treated samples to the enzymatic activity for control samples incubated without trypsin.

The ability of an enzyme to resist protease digestion is critical to the realization of novel enzyme-based biotechnologies. The susceptibility of free and immobilized enzymes to protease digestion was systematically investigated using trypsin as a model protease. Stock solutions of free HRP and free THRP were prepared at a concentration of 0.5 μg/mL, and purified HRP-AuNP and THRP-AuNP bioconjugates were prepared with saturated levels of the enzyme. Trypsin was added to each sample, and the mixtures were incubated at 37 °C. Trypsin is a proteolytic enzyme that cleaves the peptide bond at the carboxyl end of arginine or lysine in a protein. Thus, trypsin treatment of samples is expected to digest HRP and THRP, eliminating their catalytic activity toward ABTS oxidation. The enzymatic activity of the free HRP, free THRP, HRP-AuNP bioconjugate, and THRP-AuNP bioconjugate was tested after trypsin exposure for 1, 2, and 18 h. Control samples without trypsin were also incubated at 37 °C. The activity for each enzyme and bioconjugate control sample, e.g., no trypsin, was recorded to benchmark the maximum enzymatic activity, taking into account any denaturation and reduced activity resulting from storage time and temperature-dependent effects. Figure 6 plots the ratio of activity for the trypsin-treated samples to the corresponding control sample without trypsin. The free enzymes were rapidly digested, losing over 60% activity after 1 h of incubation with trypsin and rendered nearly 100% inactive after 18 h of incubation in a trypsin solution. These results confirm that Traut’s modification of the lysine residues in THRP did not prevent proteolytic digestion by trypsin. Immobilization of HRP on AuNPs, through spontaneous adsorption, slowed proteolytic degradation relative to that of the free enzymes; however, trypsin effectively neutralized 90% of the HRP activity after 18 h. Interestingly, the THRP-AuNP bioconjugate retained 100% of its initial activity after 18 h of incubation in a trypsin solution. These results corroborate that the immobilization of THRP through strong Au–S interactions imparts rigidity to THRP. The inflexibility of immobilized THRP prevents trypsin attack to cleave peptide bonds. Conversely, the loosely immobilized HRP is sufficiently flexible to allow trypsin access to arginine and lysine residues.

## Conclusions

Solvent-accessible thiols present on the surface of an enzyme are a critical parameter impacting the loading and functional fate of the enzyme adsorbed onto AuNPs. Installation of thiol functional groups on HRP via chemical modification produced a thiolated HRP analog (THRP) without altering the secondary structure or biocatalytic activity relative to the native HRP protein. The stabilities of both enzymes as a function of time and temperature were statistically equivalent. HRP and THRP were observed to spontaneously and passively adsorb onto AuNPs; however, the THRP-AuNP bioconjugate resulted in 5× greater biocatalytic activity compared to the HRP-AuNP bioconjugate. Upon further investigation, these studies suggest that adsorption of HRP and THRP does not affect the activity of either enzyme, relative to the free native enzymes, for freshly prepared bioconjugates, confirming that the active sites are fully accessible, and the secondary structure surrounding the active site is not altered as a result of interacting with the AuNP. The greater activity for THRP-AuNP bioconjugates compared to HRP-AuNP bioconjugates is attributed to 5× greater enzyme loading that is facilitated by the installed thiol groups. Moreover, the THRP-AuNP conjugate exhibited greater stability than either the free enzyme or the HRP-AuNP conjugate and is ascribed to strong interactions between THRP and AuNP that enhance structural rigidity to reduce thermal and AuNP-induced denaturation. The benefit of enhanced resistance to protease digestion is also observed for the thiolated analog of HRP immobilized onto AuNPs. This study provides valuable insights into the enzyme-AuNP interface that informs the rational design of bioconjugate systems for enhanced performance in emerging nanobiotechnologies.

## Supporting Information Available

SDS-PAGE of HRP and THRP; supernatant assay to quantify enzyme loading on AuNP; sample calculation to quantify number of enzyme molecules adsorbed on AuNP; enzyme assay of sequential bioconjugate wash cycles; photographs of enzyme-mediated colored product generated from trace enzyme adsorbed to microcentrifuge tube walls; end point assays to semiquantitatively characterize enzyme adsorption to microcentrifuge tube walls; and effect of pH on enzyme adsorption (PDF)
