# Supplementary material for: Immobilization of Thiol-Modified Horseradish Peroxidase on Gold Nanoparticles Enhances Enzyme Stability and Prevents Proteolytic Digestion
Source: Langmuir. 2024 Jun 26;40(27):13957–67. doi: 10.1021/acs.langmuir.4c01180 (PMC11238584; doi:10.1021/acs.langmuir.4c01180)
Supplement: Supplementary file 1 — la4c01180_si_001.pdf [file la4c01180_si_001.pdf]

Supporting Information for:

Immobilization of thiol-modified horseradish peroxidase on gold nanoparticles enhances enzyme stability and prevents proteolytic digestion

Faith E. Breausche[1], Annelise Somerlot[1], Jason Walder[1], Kwame Osei[1], Samuel Okyem[2], and Jeremy D. Driskell[1,*]

[1]*Department of Chemistry, Illinois State University, Normal IL, 61790*

[2]*Department of Chemistry, University of Illinois at Urbana-Champaign, Urbana IL, 61801*

Sodium Dodecyl Sulfate-Polyacrylamide Gel Electrophoresis (SDS-PAGE) of HRP and THRP

A non-reducing 4× loading buffer was prepared with glycerol (40% *v/v*), SDS (8%), bromophenol blue (0.4%), and 200 mM Tris pH 6.8. The loading buffer was diluted to 1× with DI water and the HRP/THRP stocks so that the HRP/THRP stocks yielded a final concentration of 1 mg/mL. After the samples rested in a boiling water bath for 5 min, 10 μL was loaded into the well of an 8% polyacrylamide gel. Additionally, 5 μL of Bio-Rad Precision Plus Protein Dual Color Standards was plated as the reference protein ladder. The standard procedure for an SDS-PAGE was followed per usual with the running buffer at 150 V for approximately 1 h. Afterwards, the gel was placed in a staining solution (45% MeOH, 10% acetic acid, and 0.1% Coomassie blue) over night. The next day, the gel was removed from the staining solution and placed in a destaining solution (45% MeOH and 10% acetic acid) over night.

HRP and THRP were analyzed by SDS-PAGE under non-reducing conditions to confirm that installation of the thiol did not induce unwanted protein denaturation, aggregation, or dimerization via disulfide bridging (**Figure S1**). HRP appears as a single protein band at ~40 kDa, as expected for the glycosylated protein. THRP presents a major band with an equivalent mobility as the HRP sample, indicating a MW of ~40 kDa. The 100 Da increase in molecular weight for each Traut's modified lysine is not expected to be resolved by the gel. A faint, secondary band is observed in the THRP sample with a molecular weight of ~80 kDa, suggesting the formation of a few dimers, likely due to intermolecular disulfide bridging. While the dimers are not desirable, they make up a small fraction of the THRP sample, and it serves as evidence for successful installation of thiols functional groups on the THRP.

Supernatant Assay to Quantify Enzyme Loading on AuNP

The protein in the supernatant is typically measured using the BCA assay. This standard approach proved inaccurate for quantitation of HRP and THRP loading, because significant amounts of enzyme adsorbed onto the walls of low-binding microcentrifuge tubes used to form the bioconjugates, ultimately leading to an overestimation of protein loading on the AuNPs. To illustrate this challenge, a 100 µL aliquot of 10 µg/mL HRP or THRP was added to a low-binding microcentrifuge tube for 1 h and washed three times by adding fresh buffer and vortexing. After discarding the final rinse, 1-step ABTS was added to the rinsed tube to allow for enzyme-mediated color development. **Figure S2** shows the colored product formed after 30 min, establishing that both HRP and THRP adsorbed to the centrifuge tube walls, although THRP adsorbed to a greater extent.

Sample calculation to quantify enzyme loading on AuNP

Conjugate	max rate (OD/min)	[(T)HRP] (ug/mL)	(T)HRP (ug)	(T)HRP (molecules)	AuNP (mL)	[AuNP] (NP/mL)	AuNP (NP)	(T)HRP/AuNP (molecules/NP)
HRP	0.057	0.159	0.017	2.39E+11	0.1	2.60E+10	2.60E+09	**92.0**
THRP	0.298	0.667	0.073	1.00E+12	0.1	2.60E+10	2.60E+09	**386.2**

Max rate: experimentally measured

[(T)HRP]: calculated from calibration curve in Figure 4 (HRP in AuNP and THRP in AuNP linear equations)

(T)HRP (µg): mass adsorbed based on calculated enzyme concentration and volume of conjugate sample (106-109 µL)

(T)HRP (molecules): number of adsorbed molecules calculated from enzyme mass and molecular weight (44 kDa)

AuNP (mL): volume of AuNP used to prepare conjugates

[AuNP]: concentration of AuNP used to prepare conjugates

AuNP: Number of AuNPs in conjugate calculated as volume x concentration

(T)HRP/AuNP: ratio of adsorbed enzyme molecules to number of AuNPs in the conjugate suspension

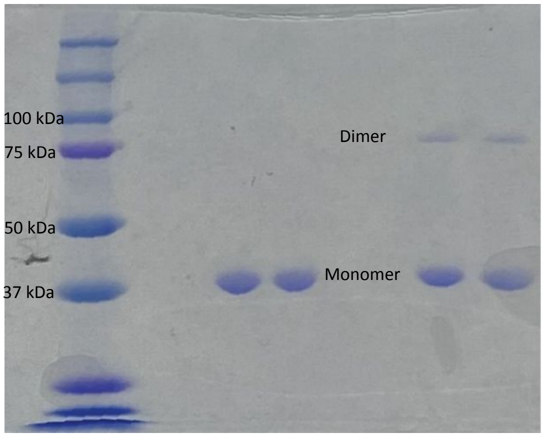

Figure S1. Characterization of HRP and THRP by SDS-PAGE. The enzymes (10 μg per well) were electrophoresed in duplicate (lane 1 = ladder, lanes 2, 5, 6 = empty, lanes 3, 4 = HRP; lanes 7, 8 = THRP.

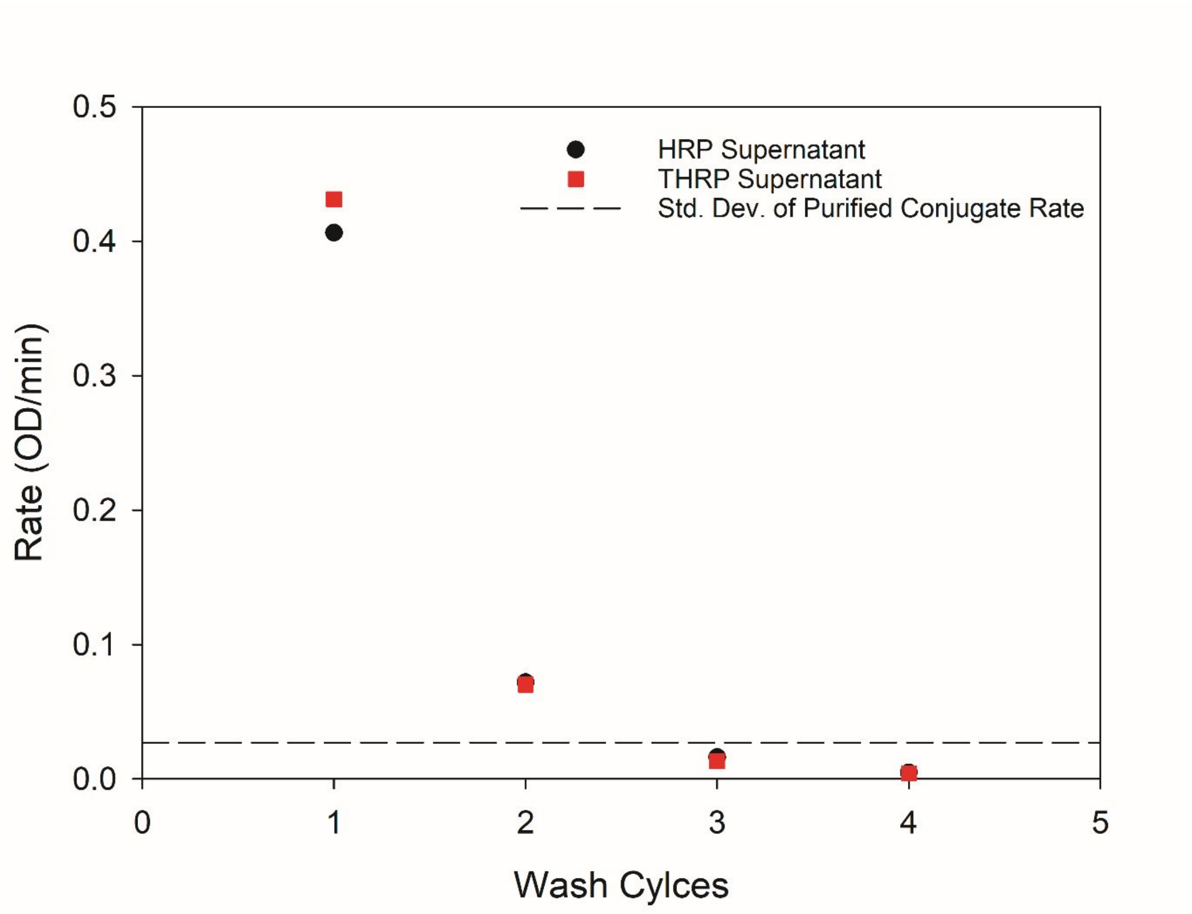

Figure S2. Enzyme activity (e.g., reaction rate) of HRP and THRP remaining in supernatant after sequential centrifugation wash cycles. The dashed line represents the standard deviation in the rate of purified HRP-AuNP and THRP-AuNP conjugates. No desorption from the purified conjugate can be detected once the rate of the supernatant is less than the variation in the bioconjugate signal.

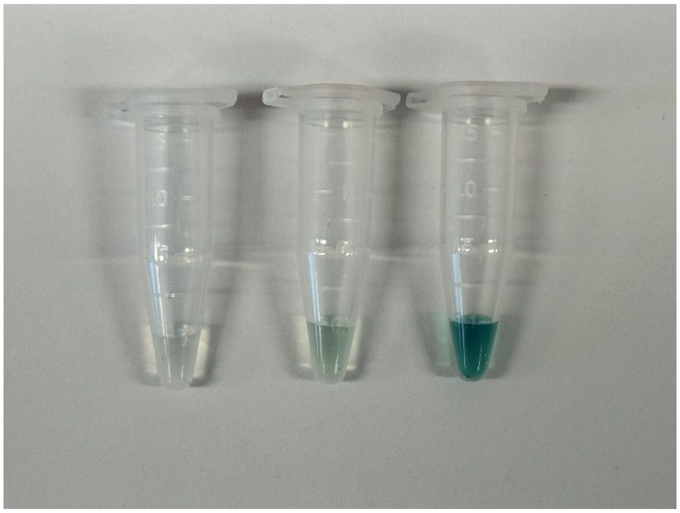

Figure S3. Photograph of endpoint assay for enzyme-mediated color formation. A 100 μL aliquot of buffer (left), 10 μg/mL HRP (center), and 10 μg/mL THRP (right) were added to microcentrifuge tubes and left at room temperature for 1 h. Samples were removed from the microcentrifuge tubes and each tube was washed three times with fresh buffer prior to the addition of 1-step ABTS. The photograph was taken 30 min after the addition of 1-step ABTS solution. The green product is a result of enzyme-mediated oxidation of ABTS from residual enzyme adsorbed to the microcentrifuge tube walls.

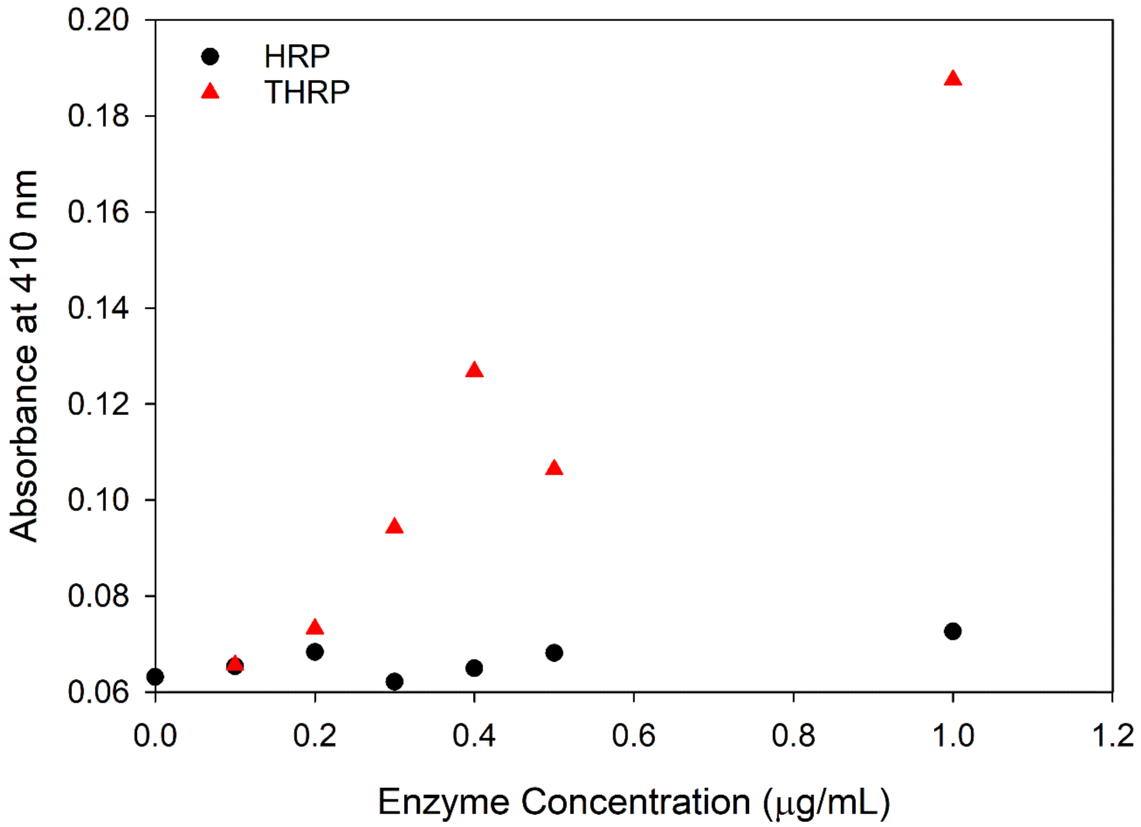

Figure S4. Endpoint assay of residual activity of HRP and THRP adsorbed to centrifuge walls. Microcentrifuge tubes filled with solutions of varying enzyme concentrations for 1 h were rinsed three times with fresh buffer, vortexing after each addition of wash buffer. Subsequently, 125 μL of 1-step ABTS was added to each microcentrifuge tube and incubated 45 min to allow for color development. Next, 100 μL aliquots were pipetted into wells of a 96-well microtiter plate and the absorbance at 410 nm was collected. Measured absorbance values correlate with enzyme adsorption to microcentrifuge walls and confirm loss of enzyme during the incubation step for conjugate formation.

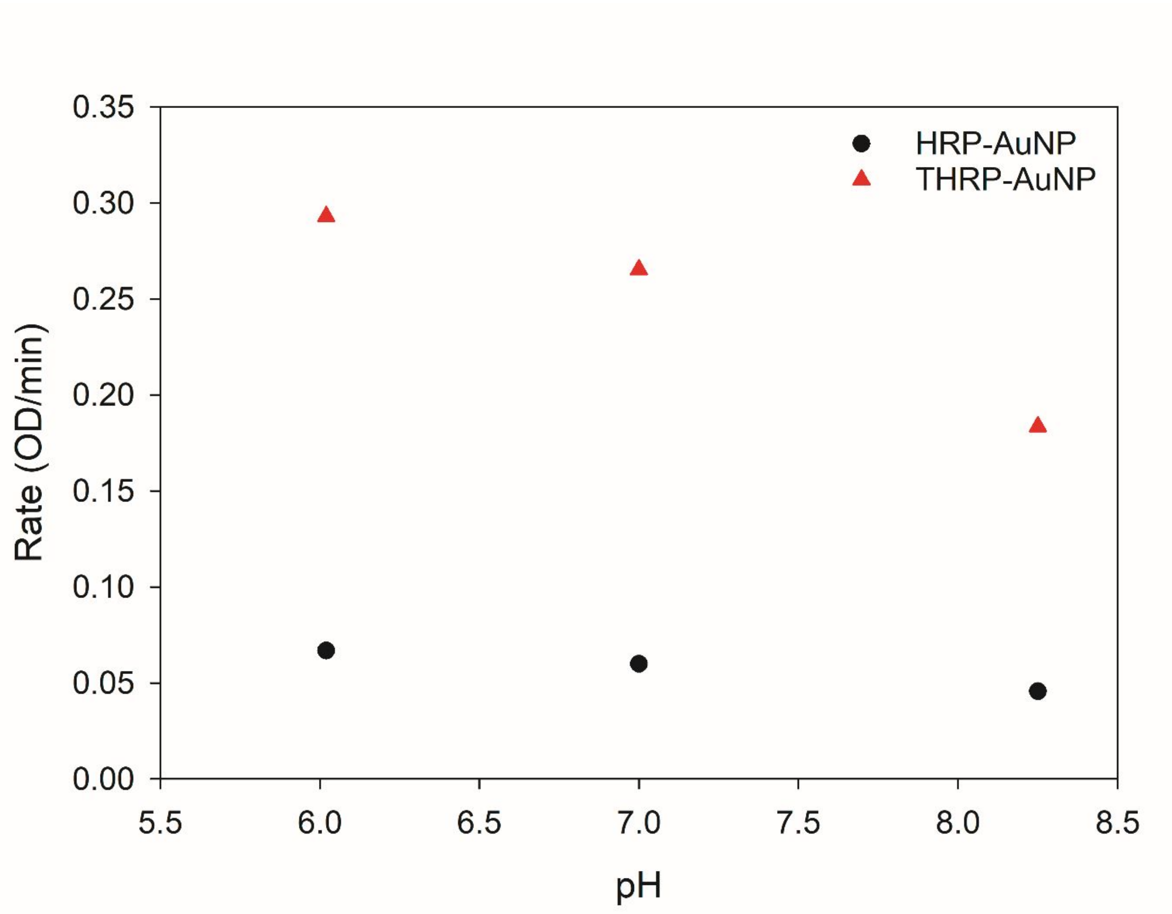

Figure S5. Enzymatic activity of HRP and THRP conjugates prepared at different pHs. Each conjugate was prepared by incubating AuNP with 10 μg/mL of enzyme for 1 h followed by purification via centrifugation prior to assessing activity.
